# Acute-on-Chronic Kidney Injury in Thyroid Hormone Withdrawal: A Case with Possible Implications for Radioactive Iodine Planning

**DOI:** 10.1155/2015/932372

**Published:** 2015-08-16

**Authors:** Elizabeth A. McAninch, Violet S. Lagari

**Affiliations:** ^1^Division of Endocrinology and Metabolism, Rush University Medical Center, Chicago, IL 60612, USA; ^2^Division of Endocrinology, Diabetes and Metabolism, University of Miami Miller School of Medicine, Endocrine Section, Bruce W. Carter Veterans Affairs Medical Center, Miami, FL 33136, USA

## Abstract

The association between renal dysfunction and hypothyroidism is of increasing clinical importance as thyroid hormone replacement may attenuate decline in renal function and improve cardiovascular outcomes in patients with chronic kidney disease (CKD). Although multiple mechanisms for the induction of renal insufficiency in hypothyroidism have been described, the renal impact of short-term, acute hypothyroidism is unknown, which has possible implications for thyroid cancer patients preparing to receive radioactive iodine (RAI). A 56-year-old gentleman with history of unilateral renal agenesis and CKD stage III presented with intermediate-risk papillary thyroid cancer. In preparation for RAI, he underwent thyroid hormone withdrawal (THW) associated with acute kidney injury (AKI), as marked by a decrease in his estimated GFR from 53 to 32 mL/min/1.73 m^2^. Upon resumption of thyroid hormone, renal function returned to baseline within months. Although AKI in this case was not otherwise associated with adverse outcome and reversed upon resumption of thyroid hormone, it is possible that this phenomenon could result in potential harm, particularly in the patient with baseline renal insufficiency. In CKD patients, preparation for RAI therapy may require special consideration; future studies should address the role of recombinant TSH to mitigate deleterious renal effects of acute hypothyroidism in this setting.

## 1. Introduction

Renal insufficiency and hypothyroidism are common conditions that may share a mechanistic relationship, as the prevalence of hypothyroidism among those with chronic kidney disease (CKD) is increased [1]. Despite consistent epidemiologic evidence, the nature of this association is not completely understood due to complexities in thyroid function interpretation in the setting of uremia and the subtleties of interaction between direct (e.g., reduced thyroid hormone signaling at the level of the nephron) and indirect (e.g., systemic hemodynamic alterations) consequences of hypothyroidism on the kidney [1, 2]. This topic has drawn particular interest in recent years as growing evidence suggests that overt or subclinical hypothyroidism could represent a therapeutic target to improve cardiovascular outcomes [1] and/or disease progression in CKD [3, 4].

One instance in which hypothyroidism is intentionally induced is in preparation of thyroid cancer patients for receipt of radioactive iodine (RAI) ablation. In this setting, patients undergo either thyroid hormone withdrawal (THW) to stimulate endogenous TSH or injection with recombinant human thyrotropin (rhTSH). Clinical guidelines state that rhTSH should be chosen for patients “unable to tolerate” hypothyroidism, although comorbidities to which this applies are not specified [5]. For instance, cardiovascular disease and psychiatric illness may represent indications for rhTSH as hypothyroidism can exacerbate these conditions [6]. Here we describe a case in which THW was associated with acute-on-chronic kidney injury.

## 2. Case Presentation

**Figure 1:**
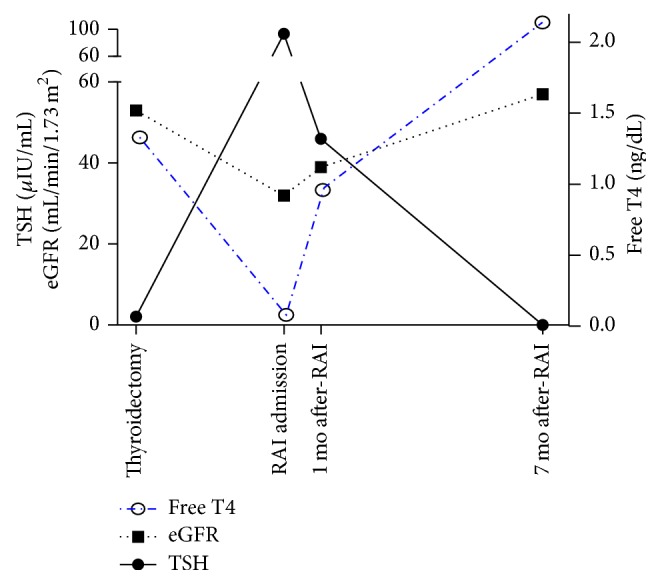
Time course of renal decompensation associated with acute hypothyroidism in levothyroxine withdrawal. At the time of thyroidectomy, the patient had CKD stage III with a normal TSH (0.27–4.2 uIU/mL), normal free T4 (0.93–1.70 ng/dL), and an eGFR of 53 mL/min/1.73 m^2^. After withdrawal of levothyroxine in preparation for RAI therapy, the TSH increased to 93 *μ*IU/mL corresponding to a decrease in the free T4; this was accompanied by an acute decrease in the eGFR to 32 mL/min/1.73 m^2^. After restarting levothyroxine to achieve TSH suppression for management of his intermediate-risk papillary thyroid cancer, the eGFR trended back toward baseline and was restored by 7 months of thyroid hormone replacement.

A 56-year-old, Caucasian male with history of uncontrolled hypertension, obesity, and congenital absence of the left kidney presented with submandibular mass. On neck ultrasound, a submandibular lipoma was demonstrated, as well as a thyroid incidentaloma. He had no family history of thyroid cancer or personal history of radiation exposure. FNA of the ill-defined, hypoechoic, 1.9 × 1.2 × 1.2 cm nodule was suspicious for papillary thyroid cancer (PTC). As part of his preoperative evaluation, lab tests showed Na 144 mmol/L, K 4.1 mmol/L, HCO_3_ 33 mmol/L, Cl 103 mmol/L, BUN 16 mg/mL, Cr 1.38 mg/dL, eGFR 53 mL/min/1.73 m^2^ (by the Modification of Diet in Renal Disease (MDRD) Study equation), glucose 85 mg/dL, TSH 2.08 *μ*IU/mL, and free T4 1.33 ng/dL (Figure 1). His only medication was amlodipine, which had been initiated 1-2 months prior to presentation; blood pressure at his preoperative visit on amlodipine 10 mg daily was 145/80 mmHg. He had not seen a nephrologist since childhood and had not previously been compliant with antihypertensives; no prior lab tests were available due to infrequent healthcare encounters.

The patient underwent total thyroidectomy without complication; pathology report showed multifocal PTC with the largest focus being 1.6 cm, surgical margins free of tumor, and focal lymphovascular invasion (<4 vessels) and with variants including diffuse sclerosing, follicular, and classical variants. None of 6 lymph nodes examined contained carcinoma. Thus, TNM stage was pT1b, pN0, and pMn/a. He was considered to be at intermediate risk for recurrence and therefore deemed a candidate for remnant ablation with RAI.

In preparation to receive the RAI, which required inpatient administration, he was started on a low-iodine diet and was withdrawn from levothyroxine. Specifically, four weeks before the planned admission, his levothyroxine was discontinued, liothyronine was started, and the low-iodine diet was initiated. Two weeks later, the liothyronine was discontinued. Lab tests were checked about one week after discontinuation of liothyronine (three weeks after discontinuation of levothyroxine and three weeks after starting the low-iodine diet); lab tests showed TSH 93.58 *μ*IU/mL, free T4 0.08 ng/dL, thyroglobulin (TG) <0.20 mg/mL, anti-TG antibodies 21.2 IU/mL (<20), and 24-hour urine iodine 68 mcg (75–500). As his TSH was at goal (>30), arrangements for admission were finalized.

On the day of admission, the patient complained of fatigue, cold intolerance, and constipation. He denied muscle pain, weakness, or change in urine output. He denied recent illnesses, use of anti-inflammatories and supplements, and any other changes to his medication regimen; he reported normal per oral intake of fluids and denied dysuria, hematuria, or renal colic. His BMI was 34 kg/m^2^, BP 94/67 mmHg, HR 53 bpm, *T* 37.0°C, and *R* 12/min. Cardiac, pulmonary, and abdominal exams were normal and he had 1+ pitting edema of the bilateral lower extremities. Neck showed healing surgical incision without erythema or discharge. Immediately prior to the RAI ingestion, “routine” admission lab tests were ordered by the admitting physician and showed serum Na 138 mmol/L, K 5.3 mmol/L, HCO_3_ 18 mmol/L, Cl 101 mmol/L, BUN 17 mg/mL, Cr 2.12 mg/dL, and eGFR 32 mL/min/1.73 m^2^ (Figure 1). He received 107 mCi of ^131^I and was discharged three days later on levothyroxine 100 mcg daily. Lab tests on discharge showed BUN 16 mg/mL, Cr 2.02 mg/dL, eGFR 34 mL/min/1.73 m^2^, and K 4.8 mmol/L. Post-RAI whole body scan demonstrated uptake in the neck consistent with thyroid remnant versus lymphadenopathy.

One month after discharge, blood pressure was 116/76 and repeat lab tests showed TSH 46 *μ*IU/mL, free T4 0.96 ng/dL, BUN 25 mg/mL, Cr 1.82 mg/dL, and eGFR 39 mL/min/1.73 m^2^ (Figure 1); levothyroxine was increased to 200 mcg daily. Six months later the blood pressure was 123/70 and lab tests showed TSH 0.060 *μ*IU/mL, free T4 2.14 ng/dL, BUN 21 mg/mL, Cr 1.29 mg/dL, eGFR 57 mL/min/1.73 m^2^, TG <0.2 mg/mL, TGab 12.5 IU/mL, HgbA1C 5.2%, 25-hydroxy vitamin D 65 ng/mL, and PTH 30 pg/mL (Figure 1). Lab tests remained similar with an eGFR ranging from 57 to >60 mL/min/1.73 m^2^ and suppressed TSH over the next year. Renal ultrasound showed absence of the left kidney with compensatory hypertrophy of the right kidney, measuring 14.1 cm, with normal echogenicity.

## 3. Discussion

Renal insufficiency is a well-described consequence of severe hypothyroidism; the mechanism, although not clearly defined, is likely multifactorial and includes direct effects on renal tubular function, which can manifest as hyponatremia caused by impaired free water clearance, decreased renal perfusion due to hemodynamic changes, and decreased glomerular filtration [2]. In addition, indirect causes have been described, including reports of hypothyroidism-induced rhabdomyolysis resulting in renal failure [7]. Although a weakness of this case is that no further work-up was performed to elucidate the specific etiology of his acute renal decompensation (e.g., urine protein quantitation or serum creatine phosphokinase), the key point remains that acute hypothyroidism was associated with acute-on-chronic kidney insufficiency that reversed upon restoration of thyroid hormone levels.

Interestingly, this patient's acute renal decompensation was only discovered incidentally after renal function tests were checked at admission during the acute hypothyroid state prior to RAI receipt. Although there is no indication that such lab collection should be considered “routine” during RAI preparation, this phenomenon may have been otherwise overlooked. The prevalence of acute kidney injury (AKI) in patients with underlying CKD undergoing THW is unknown; this is the first such reported case to our knowledge. Two cases have been reported in which THW induced AKI in patients with normal serum creatinine at baseline [8, 9], where one patient had a history of diabetes [8] and the other a history of bowel resection predisposing him to volume depletion and rhabdomyolysis [9]. Additionally, one prospective crossover study has evaluated renal function in THW and rhTSH preparation in patients with normal baseline renal function and found that while serum creatinine levels did increase in THW, creatinine levels remained within the normal range and thus this increase was not likely clinically significant [10]. However, in another study, a minority of patients with normal baseline creatinine levels did develop elevations in creatinine above the normal range during THW, but again these changes reversed upon restoration of euthyroidism [11]. It would be interesting to evaluate the population of thyroid cancer patients to determine the changes in renal function during THW; differentiating between changes in patients with and without baseline CKD would be important.

This case may hold clinical significance for thyroid cancer patients being prepared for RAI. Although the guidelines imply that patients with comorbidities potentially exacerbated by hypothyroidism should be considered for rhTSH [5], perhaps such indications should be clarified and, possibly, include CKD if these findings can be confirmed in larger studies. In this case, renal function returned to baseline after resumption of thyroid hormone, but whether such reversibility is invariable remains to be determined.
